# Effectiveness of Finnish SISU training in enhancing prehospital personnels’ work performance: A randomised controlled pilot study

**DOI:** 10.1186/s12873-025-01235-7

**Published:** 2025-05-16

**Authors:** Hanna Vihonen, Janne Karvonen, Harri Gustafsberg, Juha-Matti Huhta, Heidi Kangasniemi, Timo Jama, Sanna Hoppu

**Affiliations:** 1 Department of Emergency Medicine Services, Wellbeing Services County of Päijät-Häme, Lahti, Finland; 2 Department of Emergency Medicine Services, Wellbeing Services County of Kymenlaakso, Kotka, Finland; 3 International Performance Resilience and Efficiency Program, Pirkkala, Finland; 4 Research, Development and Innovation (RDI) Activities, Police University College, Tampere, Finland; 5 Emergency Medicine Services, Centre for Prehospital Emergency Care, Pirkanmaa Wellbeing Services County, Tampere, Finland; 6 University of Helsinki, Helsinki, Finland

**Keywords:** Prehospital personnel, Stress, Resilience, Simulation, Situational awareness, Decision-making skills, Pre-emptive

## Abstract

**Background:**

Resilience means coping with and recovering quickly from adversities. This is a highly beneficial quality for prehospital personnel, who encounter many unforeseen stressors while on duty. This study investigated whether a novel pre-emptive resilience coaching programme, ‘Finnish SISU training’ (hereafter SISU), based on the validated International Performance Resilience and Efficacy Program (iPREP), would improve the work performance by enhancing situational and decision-making skills of prehospital personnel. ‘Sisu’ is a Finnish word meaning the combination of toughness and resilience.

**Methods:**

This randomised controlled pilot study was conducted in Päijät-Häme, Finland. The sample comprised 16 paramedics, divided equally between the intervention and control groups. SISU was administered to the intervention group. Three full-scale simulation scenarios were then conducted. A blinded observer evaluated the participants’ situational awareness and decision-making skills using a structured observer form, awarding them a maximum of 10 points. Participants completed a self-evaluation form before and after each simulation scenario and the responses were rated on a 5-point Likert scale. The results of these forms were compared between groups. We also compared the median values of heart rate variability (HRV), maximum heart rate, and respiratory rate between the groups.

**Results:**

After 16 h of pre-emptive SISU, the intervention group improved their situational awareness and decision-making skills in the third simulation scenario (observer form results: intervention group median 10 [IQR 9–10] and control group median 6 [IQR 5–7], *p* ≤ 0.01). In contrast, observer ratings of the control group showed a diminishing trend in work performance across the three simulation scenarios. Self-evaluation revealed increased confidence in work performance in both study groups, in contrast to the blinded observer findings. Regarding HRV, the intervention group in contrast to the control group, recovered in minutes following the simulation scenarios, especially after the third simulation scenario (third defusing session: intervention group median HRV 27 [IQR 21–28], control group median HRV 21 [IQR 17–22], *p* < 0.01).

**Conclusion:**

SISU improved work performance, which was measured by situational awareness and decision-making skills under stressful conditions. Resilience, a skill gained from this novel training, may have positive effects on coping with stress.

**Trial registration:**

ISRCTN10221308. Registered at 19.3.2024. Retrospectively registered. https//www.isrctn.com/ISRCTN10221308.

**Supplementary Information:**

The online version contains supplementary material available at 10.1186/s12873-025-01235-7.

## Background

Situational awareness is crucial for decision-making among first responders, such as prehospital personnel and law enforcement officers as they may encounter many unknown quickly evolving situations while on duty. It involves perceiving ongoing situations and anticipating how the situation may develop.

Moreover, personality may influence interpretation and action in a situation, but even perfect situational awareness does not guarantee optimal action [1]. Therefore, an action can be beneficial or harmful regardless of situational awareness [1, 2]. Situational awareness may also differ among occupations and positions. For example, the activities occurring in emergency care situations may require a slightly different premise compared to police encounters; thus, situational awareness is likely to comprise slightly different elements.

Stress affects situational awareness and the ability to make observations and act accordingly. This is due to the physiological response of the body. The hypothalamus-pituitary-adrenal axis is activated and adrenal and cortisol is secreted along with activation of the sympathetic nervous system. As one part of the stress reaction, the thinking process of the brain shifts from the rational frontal cortex area to the inner, faster and more intuitively thinking brain areas [3]. Moreover, the ability to understand a situation is challenging if little critical information is received about it or if understanding is suboptimal or incorrect.

Resilience is the capacity to withstand or recover from adversities. A resilient individual has the ability to adapt to the emotional, physical, and mental challenges of life experiences by responding with behavioural flexibility [5]. Like situational awareness, resilience is a trainable skill [6].

In a randomised controlled study, Andersen and Gustafsberg investigated whether the validated International Performance Resilience and Efficiency Program (iPREP) could be directly integrated into training of a Finnish special police unit, and whether it could improve police performance and safety during realistic critical incident scenarios. The study used blinded observers, self-evaluation, and physiological parameters. Results revealed that officers in the intervention group displayed significantly enhanced situational awareness and overall performance and made a greater number of correct decisions [4].

Law enforcement and military personnel have incorporated resilience training in their exercises. However, for health care and emergency care personnel, such techniques remain fairly unfamiliar and are used less in practice. Hence, it is necessary to investigate how well resilience training may improve prehospital personnel’s work performance.

The primary aim of this study was to investigate whether ‘Finnish SISU training’ (hereafter SISU) improved work performance by increasing situational awareness and decision-making skills. SISU is based on the iPREP and uses breathing techniques, a positive mindset, and knowledge of the physiological and cognitive stress response. The primary measures were blinded observer ratings (maximum of 10 points); self-ratings, which were compared with observer ratings; and three physiological parameters: maximum heart rate, heart rate variability (HRV), and respiratory rate. We used HRV to achieve the secondary aim of determining how SISU affected recovery after stressful simulation scenarios.

## Methods

### Study design

This was a prospective, randomised, controlled pilot study conducted at Päijät-Häme Central Hospital Emergency Medical Service (EMS). The study was approved by the Ethics Committee of Helsinki (HUS/3235/2023) and registered in the ISRCTN registry (ISRCTN10221308). The study was conducted in accordance with the CONSORT statement [5].

### Population

Päijät-Häme, a Finnish region with 200,000 residents, operates a unified three-tiered EMS system with basic life support, advanced life care, and a physician-manned ground unit on duty from 08:00 to 20:00. The EMS employs prehospital physicians, paramedics, nurse paramedics, and expert critical care paramedics. Additionally, an EMS field commander coordinates and leads operations during major incidents in the operative field and adjusts the dispatch centre calls to local ambulance availabilities and detailed preferences.

### Recruitment

In the focal EMS system, prehospital physicians are often dispatched along with paramedics to attend to critically ill patients. We thus included prehospital physicians in the study. In June and July 2023, 20 paramedics and 4 prehospital physicians were recruited. Informed consent was obtained from all participants. Paramedics with a minimum of three years’ clinical experience in EMS and physicians with at least one year of operative EMS experience were eligible. We excluded individuals who were aged under 18 or pregnant, had already participated in iPREP training or a similar coaching programme, or used cortisone-based allergy medication during the study period or two weeks prior.

### Core components of SISU based on the iPREP

As noted above, the iPREP has been studied as an intervention technique for enhancing Finnish police officers’ psychological and physiological control during stressful critical incidents [4]. Its core components include (a) education about the physiology of the stress response system, energy management, and fuelling for peak performance; (b) group instructions on using mental focus and visualisation to enhance sensory perception and situational awareness in performance and non-performance settings; and (c) instruction and use of biofeedback to practice controlled breathing exercises that have been shown to enhance central nervous system control during stress [4]. SISU has the same core elements as the iPREP, with minor adaptations focusing more on the working environment of healthcare personnel, as well as education concerning cognitive biases that affect work performance (Additional file 1).

### Randomisation

After enrolment, randomisation was performed using a local EMS shift coordinator blinded to the study protocol. The coordinator allocated free time between shifts for the study participants, listed their names in a random order, assigned participants to two groups, and finally subdivided each group into two teams of four. Beyond this assignment role, the shift coordinator did not participate in the study or receive SISU or iPREP training. On each simulation day, both teams in the intervention group and the control group separately completed the same simulation scenario. The blinded observers had no knowledge of which group was performing the simulation.

All study participants received pseudonyms during the simulation scenarios in accordance with the Data Protection Act. Each team had one fully equipped ambulance and one training ambulance at their disposal. All study participants were fully equipped with personal work clothing and gear, as well as a communication device with specified radio frequencies.

### Study intervention

The intervention group underwent two full days of SISU. Participants were not pre-assessed for resilience skills or homogeneity. Pre-emptive resilience training was conducted by an accredited Mind Coach, the official title awarded through the Finnish stress management coach training based on the iPREP method. The Mind Coach was also a member of the research team. The control group followed a normal daily routine prior to the simulations.

Within ten days of completing SISU, all study participants engaged in three full-scale simulation scenarios arranged on three consecutive days, one simulation per day, with simulation settings as authentic as possible, including unfamiliar apartments with volunteer actors. The simulation scenarios were designed to emphasise the need for good situational awareness and decision-making skills in a ‘high-stress environment’. At a preliminary briefing, participants were informed that the simulation scenarios may be stressful but not more so than possible encounters while on duty. Participants were instructed to perform as they would normally work.

Before the simulation scenarios, participants completed a self-evaluation form, responding on a five-point Likert-scale (1 = very insecure, 5 = very confident) to the following statements: ‘*I feel confident and will manage the simulation scenario well*’; ‘*I consider myself to have good situational awareness*’; ‘*I am able to make decisions easily*’; and ‘*I believe it will be easy to resume other duties after the simulation*’ (Additional file 2).

During the simulation scenarios, observers with a paramedic background, no experience of iPREP or SISU training, and no other role in the study completed a structured observer form. This form was designed to measure participants’ situational awareness and decision-making skills during the simulations (Additional files 3–5). Observers were blinded to the study groups (intervention vs. control). As situational awareness precedes decision making, on the observer form, a maximum of 10 points were awarded for situational awareness and decision-making [2]. Observers had 10 min to familiarise themselves with the form before the simulation. After each simulation scenario, the observers gathered to discuss their findings. All simulation scenarios were recorded on camera so that the observers could individually review their real-time observations over the next 10 days to ensure accurate ratings. To avoid bias, observers and participants were separated during and after the simulation scenarios, until the final observer forms were completed. After the three simulation scenarios were completed, the research team members compared the observer ratings, self-ratings, and physiological parameters of the study groups.

Participants attended a mandatory defusing session run by a professional counsellor after each simulation scenario. Only the counsellor and four team members were present during the defusing session. During this session physiological parameters continued to measure and record using Firstbeat Life (Jyväskylä, Finland) and relayed to the research team under pseudonyms [6]. The defusing session’s main purpose was to ensure the mental wellbeing of participants after each stressful simulation scenario and to record the physiological measures (maximum heart rate and HRV).

### Study setting

The starting point for all simulation scenarios was when the team of participants arrived at the scene by ambulance and opened the door. Participants first met the research team members and observers at a gathering point away from the simulation scene. The self-evaluation form was completed before the simulation scenario, and the observer manually measured participants’ respiratory rate. Subsequently, participants received the initial dispatch call information, including the address of the simulation scenario, via a communication device. They were required to drive a short distance to the simulation site, while the observers walked there with the research team.

The first simulation scenario involved a case of intoxication with a surprise element: a father brandishing a gun (Additional file 6). The second scenario comprised an old woman with dementia experiencing severe stomach pain, with several distractions at the scene (Additional file 7). The third scenario involved a trauma patient whose jet ski had collided with an underwater rock; the local volunteer lake rescue service offered aid (Additional file 8). After each simulation scenario, participants completed the self-evaluation form again, and their respiratory rate was manually measured by the observer.

### Outcomes

The primary outcome was the impact of SISU on situational awareness and decision-making skills, as a marker of gained resilience. This was measured by observer ratings and self-ratings, and a comparison between the two. We also measured the median physiological values of HRV, maximum heart rate, and respiratory rate The secondary outcome was how training affected recovery after the simulation scenarios.

### Data collection

Data were collected manually from self-evaluations and observer forms using participant pseudonyms. All data were scanned, analysed, and stored on a secure computer to which only the corresponding main authors had access.

Physical parameters were recorded manually and by using the scientifically validated Firstbeat Life device [6]. HRV is the variation in time between consecutive heart beats in milliseconds. Firstbeat Life calculates HRV from the square root of the mean squared differences of successive intervals, *NN50*; the number of interval differences of successive NN intervals greater than 50 ms; and the proportion derived by dividing *NN50* by the total number of NN intervals, *pNN50*. NN means ‘normal-to-normal’, and NN intervals are those between adjacent QRS complexes resulting from sinus-node repolarisation [7].

HRV can be used to express the balance between the sympathetic and parasympathetic nervous systems. In the sympathetic state, HRV decreases; in the relaxed parasympathetic state, HRV increases. Higher HRV can be used as an index of resilience [8].

### Statistical analysis

Categorical data were analysed using Fisher’s exact test. Non-categorical data were analysed using the Mann-Whitney U test. Correlations were analysed using Spearman’s correlation coefficients. Mean and standard deviation (SD are reported for normally distributed variables, while median and interquartile range (IQR) are reported for skewed variables. As this was a pilot study, a power analysis for an effective sample was not performed. The sample size was the same as that used in the aforementioned study with Finnish police officers [4]. All statistical analyses were performed using GraphPad Prism 10 for Mac OS X (GraphPad Software, San Diego, CA).

## Results

### Demographics

**Fig. 1:**
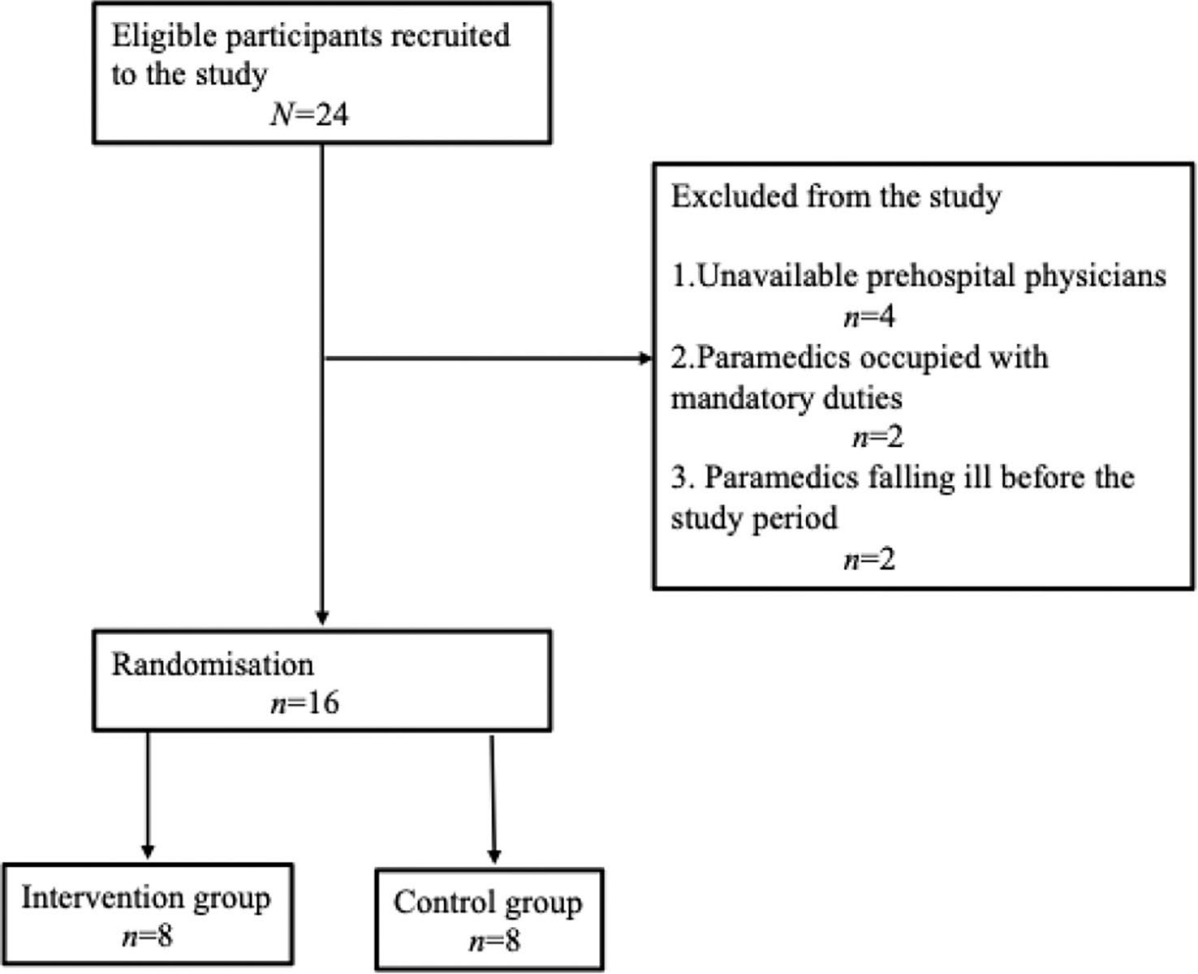
Consort flow diagram

The four recruited prehospital physicians had insufficient time between operational shifts to participate in this study. Therefore, the final sample comprised 16 paramedics (Fig. 1). Participants’ characteristics are listed in Table 1.

**Table 1:** Demographics of study participants

	All	Intervention group	Control group	*p*
Age (years), median IQR	36 (33–41)	36 (32–39)	39 (34–43)	0.43
Sex, female (%)	11 (69)	6 (75)	5 (63)	> 0.99
Work experience (years), median IQR	5 (3–16)	4 (3–14)	9 (4–17)	0.19

### Blinded observer findings

**Fig. 2:**
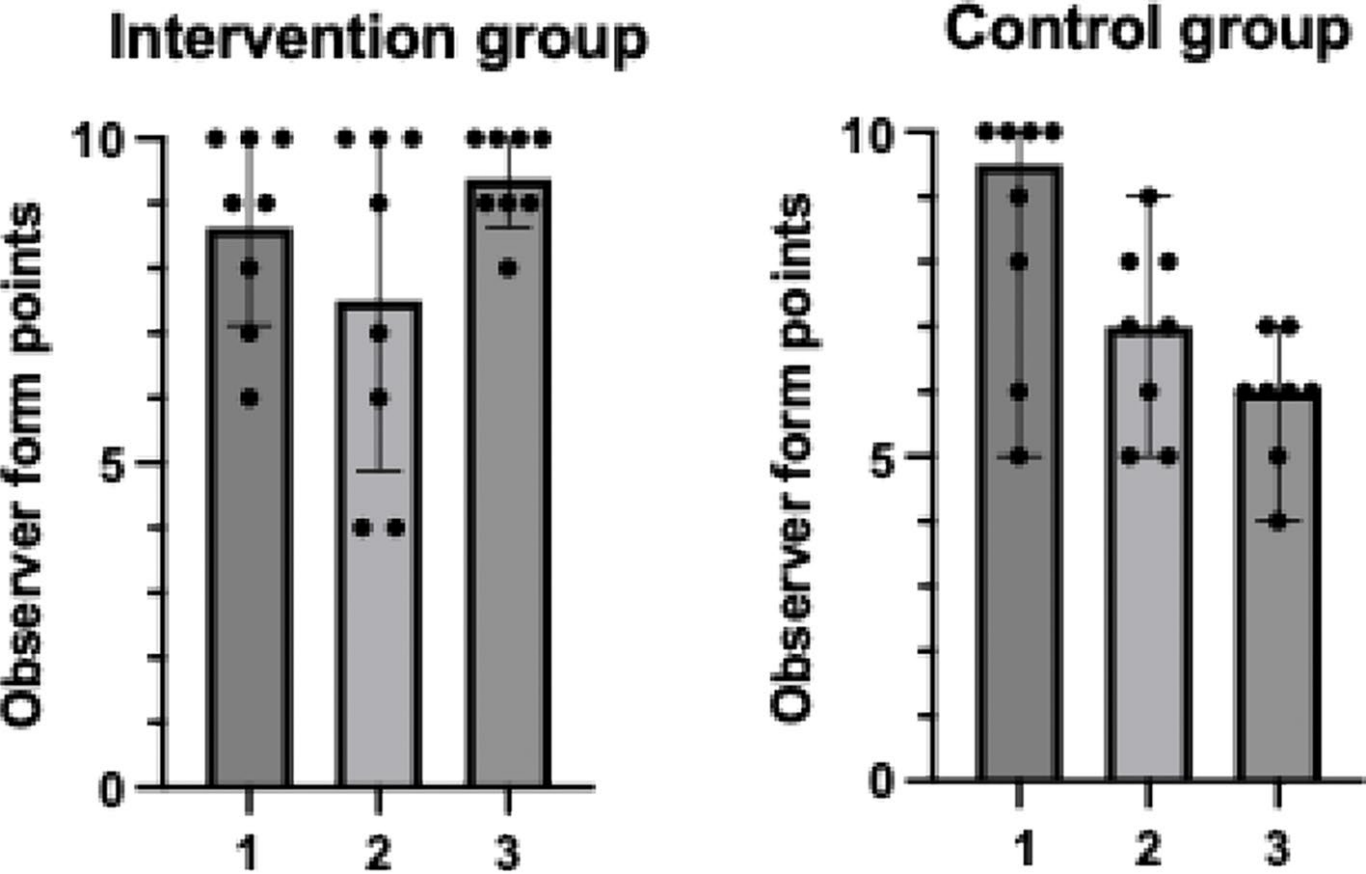
Comparison of median with interquartile range of combined situational awareness and decision-making skills as rated by the blinded observer. 1 = simulation scenario 1 (*p* = 0.96); 2 = simulation scenario 2 (*p* = 0.55); and 3 = simulation scenario 3 (*p* ≤ 0.01)

The observers’ findings revealed that the intervention group maintained situational awareness and decision-making skills in the third simulation scenario, despite receiving only 16 h of SISU with intervention group median 10 [IQR 9–10] and control group median 6 [IQR 5–7] respectively, *p* ≤ 0.01(Fig. 2). In contrast, the control group showed a diminishing trend in situational awareness and decision-making skills over the three consecutive study simulation days (Fig. 2).

### Self-evaluation findings

Self-evaluation forms were completed before and after the simulation scenarios. Participants in both groups reported being prior the simulation scenarios either confident or very confident (4 or 5 on the Likert scale) The study participants also estimated that they maintained good situational awareness and decision-making skills (Likert scale score of 4 or higher) in each simulation scenario. A comparison of the self-evaluation results between the intervention and control groups is shown in Additional file 9. The differences were not statistically significant.

### Physiological findings

The physiological results demonstrated that all study participants were physically stressed during the simulation scenarios. Participants in the intervention group had a higher median maximum heart rate in all simulation scenarios, especially in the third scenario (intervention group median 139 [IQR 135–153], control group median 130 [IQR 116–136], *p* = 0.04) (Additional file 10).

**Fig. 3:**
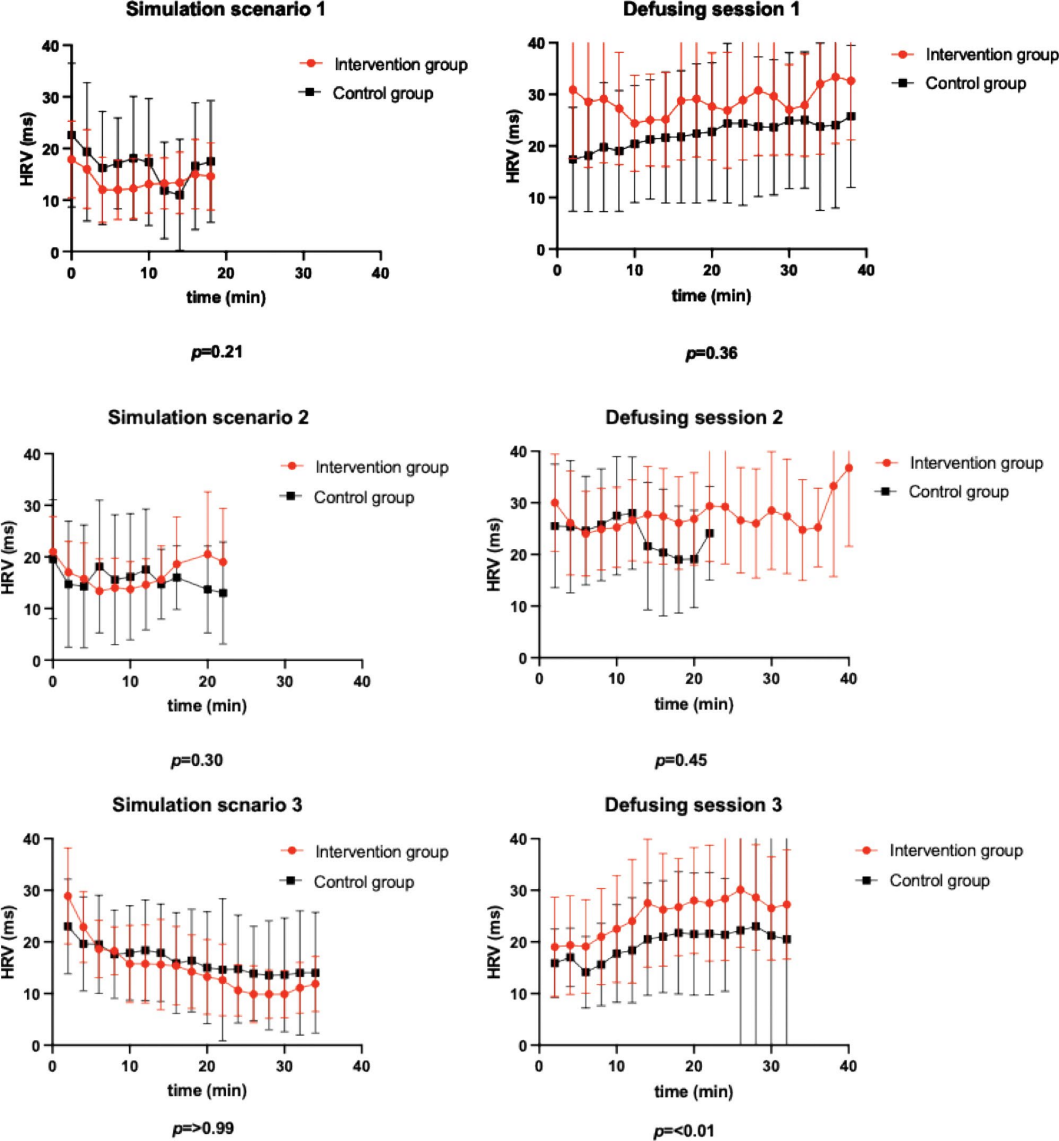
Comparison of median HRV trends and interquartile ranges during simulation scenarios and subsequent defusing sessions. HRV = heart rate variability, ms = milliseconds, min = minutes

During the simulation scenarios, participants in the intervention group tended to have lower median HRV than the control group participants (Fig. 2). By contrast, in the immediate defusing sessions after simulation scenarios, the intervention group displayed a trend of increased median HRV, particularly after the third simulation scenario (third defusing session: intervention group median HRV 27 [IQR, 21–28], control group median HRV 21 [IQR, 17–22], *p* < 0.01) (Fig. 3).

## Discussion

The main finding was that participants who underwent SISU showed improved situational awareness and decision-making skills, particularly in the third simulation scenario. Both study groups reported high self-confidence in their situational awareness and decision-making skills before and after the simulation scenarios. Furthermore, HRV data showed that in comparison to the control group, the intervention group recovered faster in minutes during the defusing sessions, particularly after the third simulation scenario.

### Improved situational awareness and decision-making skills

The intervention group may have used SISU to regulate their thought processes. When experiencing the triggering emotions of stress, study participants may intentionally have slowed their breathing patterns. Indirect control of the autonomic nervous system can result in the modulation of carbon dioxide levels and sinus arrhythmia, which slows the heart rate during exhalation [9–12]. Physiologically, the brain’s thought processes revert to the slower conscious frontal cortex area [13–15]. Without SISU, control group participants may have been more susceptible to nonconscious thought processing and cognitive biases under pressure.

Despite feeling confident initially, control group participants experienced increasing stress with each simulation, leading to declining situational awareness and decision-making skills over time, which is indicative of cumulative psychological stress. Breathing techniques and mental exercises are effective for enhancing performance in stressful situations [16, 17]. For the intervention group, SISU employed mental exercises to prepare for and immediately recover after a simulation scenario. Our training focused on gratitude, meaning, and self-compassion—all shown to have positive results in previous human performance studies [18, 19]. Breathing techniques and mental exercises have been shown to improve performance under pressure and are used by law enforcement and military personnel; however, healthcare and emergency care personnel are less familiar with them [4, 20–24].

### Self-evaluation insights

Self-evaluations indicated a blind spot in thought processes due to brain short-circuiting and biased thinking of which participants were unaware. Anticipating stress and interpersonal conflicts had minimal impact on participants’ performance perceptions. For participants in both groups, high self-confidence in their situational awareness and decision-making skills was reported before each simulation scenario and remained unchanged thereafter.

### Physiological insights

Physiologically, the intervention group showed higher stress markers (higher maximum heart rate and lower HRV), indicating higher mental alertness, and possibly a flow state. Additionally, the HRV results showed that the intervention group recovered faster than the control group after all three simulations.

### Acute stress may accumulate to chronic stress

Work-related stress may accumulate, which is known to cause mental disorders and physiological changes in the body [25, 26]. Healthcare workers and armed police officers are at increased risk [27]. Our finding of faster recovery in the intervention group indicates that pre-emptive resilience training may prevent cumulative chronic stress and improve long-term work performance and even patient safety.

### Value of using blinded observers

This study was inspired by previous research on police resilience [4]. To our knowledge, only one prior study has explored this topic among emergency medical personnel; unlike our research, it did not use blinded observers. That study was a multicentre randomised controlled trial conducted in Chicago, United States, and it investigated the effects of resilience training for emergency medicine residents [28]. Residents underwent mental skills training encompassing box breathing, positive self-talk, self-visualisation of the task, and use of cue words to activate selective attention. The training method was assessed using the Spielberger State-Trait Anxiety Inventory (STAI-6) and a post-audit self-evaluation [29]. The 61 participants wore chest bands for physiological measurements (Polar Oy; Kempele, Finland). The results showed no statistically significant differences between the intervention and training groups.

Studies that employ only self-evaluation without an outside observer fail to consider the effect of psychological fatigue during stressful conditions. This causes thought processing to be directed to the faster unconscious amygdala area, which is prone to memory deficits, biases, and short circuiting of the brain. The result is a blind spot in an individual’s ability to adequately self-evaluate their performance in a stressful situation.

### Strengths and limitations of the study

The strength of our study was the use of an outside blinded observer, who was not prone to bias during the evaluation of the study participants’ performance with regard to situational awareness and decision-making skills. The limitations of this study include its small sample size and the omission of pre-assessment of participants’ resilience skills and homogeneity. Moreover, we did not inquire about intake of coffee, tobacco, or medications such as beta-blockers, which may affect the results. Furthermore, randomisation was not performed as a traditional lottery; instead, we asked the EMS shift coordinator—responsible for allocating daily work shifts—to randomly list participants, divide them into two groups, and subdivide each group into two teams of four. Hence, this randomisation process may have affected the reliability of the results. The three simulation scenarios had different narratives and were not fully standardized, which may be considered a limitation. The leisure time spent between the simulation scenarios could also have affected the study results. However, we did measure HRV three days prior and 24 h after the last simulation scenario, and the results showed that study participants were not affected by high-stress situations (unpublished data). Even though observer and self-evaluation forms were used in the prior study of Finnish police [4], these forms were not prior trained or calibrated. Hence, they lacked validation. To avoid bias in respiratory count, the observer measured this while each participant was completing the self-evaluation form. However, the stressful situation, participants wearing work gear, and observer measuring the respiratory count only once may have affected the results [30].

Future multicentre studies with larger sample sizes should validate the effectiveness of SISU for prehospital physicians and across healthcare settings, such as emergency rooms, operation theatres, and hospital wards.

## Conclusion

The SISU-trained intervention group maintained better situational awareness and decision-making skills in the third simulation scenario. This pre-emptive resilience coaching programme also facilitated recovery in minutes after the simulation scenarios. This shows the potential of SISU for positively impacting on work performance in healthcare settings by preventing cumulative stress.

## Electronic supplementary material

Below is the link to the electronic supplementary material.

Supplementary Material 1

## Data Availability

The datasets used during the current study are available from the corresponding authors upon reasonable request.

## Abbreviations

ANS: Autonomous nervous system
EMS: Emergency Medicine Services
HRV: Heart rate variability
IQR: Interquartile range
iPREP: International Performance Resilience and Efficiency Program
STAI-6: Spielberger State-Trait Anxiety Inventory
