# Supplementary material for: Effectiveness of Finnish SISU training in enhancing prehospital personnels’ work performance: A randomised controlled pilot study
Source: BMC Emerg Med. 2025 May 16;25:80. doi: 10.1186/s12873-025-01235-7 (PMC12082985; doi:10.1186/s12873-025-01235-7)
Supplement: Supplementary file 1 — Supplementary Material 1 [file 12873_2025_1235_MOESM1_ESM.docx]

**Supplementary material**

**Additional file 1**

**Finnish SISU training method**

1.Philosophical value basis

-set of values

-focus of meaning

-self-knowledge

-personality

-temperament and tendencies

-roles

-motivation

2.Physiology

-breathing and breathing technics

-stress

-the mind-body system

-autonomic nervous system

-biofeedback

3.Mind

-learning

preperation for the task

-emotional intelligence

-thinking skills

-cognitive biases

4.Awareness skills

-Situational awareness

-decision-making

-flow

-attention skills

5.After task

-after action review and redo- technic

-sense of coherence

-imagery training meaning of defusing

-technical defusing

-team philosophy

**Additional file 2**

**Self-evaluation audit form**

(1 very insecure – 2 mostly insecure – 3 does not know – 4 confident – 5 very confident)

1. I feel confident and will manage the simulation scenario well.

1 2 3 4 5

1. I consider myself to have good situational awareness.

1 2 3 4 5

1. I am able to make decisions easily.

1 2 3 4 5

1. I believe it will be easy to resume other duties after the simulation.

1 2 3 4 5

**Additional file 3**

**Observer form, simulation scenario 1**

Participant study number:_________________________

Respiratory rate before simulation_____________________ breaths/minute

Respiratory rate after simulation_____________________ breaths/minute

**Situational awareness**

1. Environmental awareness. Does the participant notice upon visual inspection and/or verbalize the condition of the apartment with empty medication packages, a whisky bottle and small children’s toys: Yes____ (1 point) No _____
2. Active or passive involvement of a preliminary survey using the cABCDEF approach Yes____ (1 point) No____
3. Appearance of the threatening father noticed Yes____ (1 point) No_____
4. Gun noticed and a rational emotion of awareness Yes____ (1 point) No_____
5. Team performance flow: smooth teamwork Yes____ (1 point) No_____

**Decision-making skills**

1. One paramedic taking leadership and tasking others in the team with different work roles

Yes____ (1 point) No ______

1. Performing roles adequately according to tasking

Yes____(1 point) No. ______

1. Non-technical crew management (CRM) communication used

Yes ____(1 point) No _____

guidance for observing non-technical communication:

- No CRM communication observed (tick “No”)
- A single closed-loop confirmation of another team member’s words (tick “No”)
- Some closed-loop confirmation of other team member’s words (tick “No”)
- Interactive closed-loop communication with eye contact and physical gestures (tick “Yes”)
- Highly interactive and systematically approached CRM communication, involving verbal and physical gestures of non-technical communication techniques, leading to joint decision making (tick “Yes”)

1. Reacting appropriately to the threatening man with the gun. Verbal or silent gesture of decision making concerning an exit-plan with regard to team safety.

Yes_____(1 point) No____

1. Overall decision making on team safety

Yes_____ (1 point) No_____

guidance for observing team-safety-related decision making:

- No sign of decision making related to team safety (tick “No”)
- A single verbal expression of decision-making on overall team safety recorded, but no action (tick “No”)
- Some verbal expression of decision-making regarding overall team safety, but no action (tick “No”)
- Interactive decision-making skills related to overall team safety; verbal and action, with equipment placement (tick “Yes”)
- Systematically interactive decision-making on overall team safety, with verbal communication of an exit plan and equipment placement, and an understanding of a possibility of the unexpected and how the team is situated in the appartement, observed as not turning one’s back from the front door that is kept open. (tick “Yes”)

**Additional file 4**

**Observer form, simulation scenario 2**

Participant study number:_________________________

Respiratory rate before simulation_____________________ breaths/minute

Respiratory rate after simulation_____________________ breaths/minute

**Situational awareness**

1. Environmental awareness. Does the participant notice and/or verbalize the condition of the apartment: food in the fridge, two home care folders on the table?

Yes_____(1 point) No______

1. Active or passive involvement of a preliminary survey with cABCDEF approach. Yes____(1 point) No______
2. The EMS field commander adds pressure via radio. Despite this, the participant remains aware of the non-urgent nature of the patient’s situation. Yes______( 1 point) ____ No____
3. Visual, gestural or verbal acknowledgement of the content of the home care folders: verbalizing which folder is the patient’s, acknowledges the recordings of dementia. Being part of joint situational awareness. Yes____(1 point) No________
4. Team performance flow: smooth teamwork. Yes____ (1 point) No_____

**Decision-making skills**

1. One paramedic taking leadership and tasking others in the team with different work roles.

Yes____ (1 point) No ______

1. Taking appropriate equipment (monitors, stretcher, keeping the patient warm, no double equipment as there is only one patient).

Yes_____(1 point) No______

1. Non-technical crew management (CRM) communication used.

Yes ____(1 point) No _____

guidance for observing non-technical communication:

- No CRM communication observed (tick “No”)
- A single closed-loop confirmation of another team member’s words (tick “No”)
- Some closed-loop confirmation of other team members’ words saying (tick “No”)
- Interactive closed-loop communication with eye contact and physical gesture (tick “Yes”)
- Very interactive and systematically approached CRM communication, involving verbal communication with this communication technique, towards decision-making (tick “Yes”)

1. Verbal or gestural acknowledgement of being part of joint decision making as a team of two paramedics; one team leaves the scene and another stays on after reaching a joint consensus that there is not to be an urgent unexpected change in the patient’s condition, as the field officer requests an available ambulance to another scene.

Yes____(1 point) No____

1. The decision to leave the patient at home has been communicated verbally or with an appropriate physical gesture. Yes____(1 point) No____

**Additional file 5**

**Observer form, simulation scenario 3**

Participant’s study number:_________________________

Respiratory rate before simulation_____________________ breaths/minute

Respiratory rate after simulation_____________________ breaths/minute

**Situational awareness**

1. Environmental situational awareness related through eye contact, physical gestures or verbalization. This is a trauma patient and a water rescue mission. Yes____(1 point) No______
2. Active communication with the lake guard front rescue. Yes_____(1 point) No______

Guidance for the observer:

- No communication (tick “No”)
- A single gesture to communicate with the lake rescue guard (tick “No”)
- Some gestures to communicate with the lake rescue guard (tick “No”)
- A single gesture of response to a suggestion of communication with the lake rescue guard (tick “No”)
- Clearly interactive communication with the lake rescue guard (tick “Yes”)
- Systematic interactive tactical planning and communication according to the patient’s situation that everyone participates in (tick “Yes”)

1. First response preparing or treatment on a boat or the ambulance, including the preparation or treatment of the patient, keeping the patient warm, stabilization of a patient with a spinal injury, as well as all other necessary ambulance preparedness for transporting a trauma patient (heating on, iv lines, etc.).

Yes_____(1 point) No__________

1. Actively or passively involved in a preliminary survey with a systematic cABCDEF approach.

Yes____(1 point) No_____

1. Team performance flow: smooth teamwork. Yes____ (1 point) No______

**Decision making**

1. One paramedic taking leadership and tasking others in the team with different work roles. Decisions made regarding who stays on the shore to prepare the ambulance and who goes along with the lake rescue guard to the patient.

Yes____ (1 point) No ______

1. Active or passive involvement by gestures of decision-making of gathering appropriate trauma patient equipment. Yes______(1 point) No_______
2. Active communication via radio phone between the paramedics on the boat and those in the ambulance on shore.

Yes_____(1 point) No_______

Observer guidance:

- None (tick “No”)
- A single piece of information with no response communicated between the boat crew and the ambulance crew (tick “No”)
- Some information with no response communicated between the boat crew and the ambulance crew (tick “No”)
  - Interactive communication, but without decision-making regarding a mutual plan (tick “No”)
- Interactive systematic communication regarding decision making, according to mutual situational awareness.

1. Decision-making of treatment according to a spinal injury trauma-patient. Active verbal, passive physical gesture, and joint treatment with the result of immobilization of the spine and keeping the patient warm with blankets. Yes_____(1 point) No______
2. Decisive decision-making regarding patient evacuation and transport to the appropriate hospital, including consultation of a prehospital physician. Yes_____(1 point) No________

**Additional file 6**

Simulation scenario 1

| **Target group:** | Päijät-Häme Wellbeing Services County, prehospital physician and paramedics  Control group (5 + 1)  Intervention group (5 + 1) |
| --- | --- |
| **Aim of the simulation:** | The purpose of the simulation is to investigate the stress reactions and actions of prehospital personnel under pressure, as well as their teamwork, ability to maintain situational awareness and decision-making.  This the first in a series of three simulation scenarios included in the study “**Effectiveness of Finnish Sisu Training, a pre-emptive resilience coaching programme in enhancing the work performance of prehospital personnel”**. |
| **Preliminary briefing:** | The study participants taking part in the simulation scenarios are briefed to conduct themselves as if they were involved in a real patient case scene.  Familiarizing the participants with the equipment on hand and making sure that it functions properly.  Checking that the VIRVE radiofrequency channels are correct.  The starting point of the simulation is established, and participants are reminded of the simulation being a part of this research and that the simulation will be a full-scale scenario. The study participant should perform as they would in a real patient case.  Participants receive research numbers. |
| **Conducting the simulation:** | FULL-SCALE  Location: apartment on Paraatikatu, Lahti, Finland  Patient assignment: serious intoxication  Prehospital care units: EPH 00, EPH 12X & EPH 12X  If the paramedic unit asks for additional information:  “Very vague situation. The caller was a man, who sounded calm. He has found his female friend unconscious on the living room floor. It seems that the female friend has drunk alcohol, the couple has argued, after which the caller has gone out to a bar. He has been out for approximately 2 hours. Some pills may have been taken.”  On the scene:  A nice tidy apartment. Wine bottles on the kitchen counter, a baby crib and toys in the apartment.  A young woman aged roughly 25 years is lying on her stomach on the floor. (The respiratory rate is the actor’s natural rate, and temperature of her skin is also the actor’s own). The man has covered his partner with a blanket.  The man is calm. (The male actor will be briefed to be calm and friendly)  Vital signs:  A: Open  B: Respiratory rate is the actor’s natural rate, SpO2 91%, respiratory sounds are symmetrical and clear (actor’s own), the patient is snoring lightly (if jaw lift is manoeuvred, the snoring will stop).  C: RR 90/60 mmHg, heart rate 98 beats/min, sinus rhythm.  D: Pupils are midsized and symmetrical, not so clearly reactive to light, the blood glucose is the actor’s natural level.  E: No signs of trauma. Temperature from the ear 37.2 °C.  Treatment:  Suspicion of bentsodiatzepine overdose, based on visual inspection of the patient and the surrounding empty medication packages.  IV line  Flumatsenile 0.2 mg i.v. with repetition. On the second dose, the patient begins to react, but does not react sufficiently.  A third older person arrives on the scene (patient’s father). He is anxious about the patient and blames her male partner (the caller). The door should be opened by the caller unless the door was left open.  The caller and the older man start to argue. The older man goes to the kitchen. When he comes back, he has a gun that bulges from his trousers. It can be seen clearly, but he does not take it into his hands.  If the paramedics notices this threatening situation, the caller shouts to not call the police. |
| **Attention:** | **Safety word to stop the simulation: Acute-repeat acute. Everyone shouts acute.**  The simulation scenario can flow spontaneously and take its own course; only the actors will be guided and the prehospital physician and prehospital personnel will be observed for research purposes. |
| **Supplies:** | Paramedic unit equipment X 2  Prehospital physician unit  Personal gear (clothes, work shoes, safety vests)  iSimulate  VIRVE x 8 (6 for trial participants)  Connection to Codea control 🡪 Dispatch message  Props: wine bottles, bentsodiatzepine packages, toy gun, make-up (the intoxication patient has smudged make-up on her face).  Camera/ camera operator  Simulation instructor  Observers  Vest with sign indicating the voluntary outside  defusing professional |
| **Defusing after the simulation:** | After the simulation scenario, a short defusing is held to make sure that everyone is alright. This does not entail any inspection on individual performance. |

**Additional file 7**

Simulation scenario 2

| **Target group:** | Päijät-Häme Wellbeing Services County, prehospital physician and paramedics  Control group (5 + 1)  Intervention group (5 + 1) |
| --- | --- |
| **Aim of the simulation:** | The purpose of the simulation is to investigate the stress reactions and actions of prehospital personnel under pressure, as well as their teamwork, ability to maintain situational awareness and decision making.  This the second in a series of three simulation scenarios included in the study “**Effectiveness of Finnish Sisu Training, a pre-emptive resilience coaching programme in enhancing the work performance of prehospital personnel”**. |
| **Preliminary briefing:** | The study participants taking part in the simulation scenarios are briefed to conduct themselves as if they were involved in a real patient case scene.  Familiarizing the participants with the equipment on hand and making sure that it functions properly.  Checking that the VIRVE radiofrequency channels are correct.  The starting point of the simulation is established, and participants are reminded of the simulation being a part of this research and that the simulation will be a full-scale scenario. The study participant should perform as they would in a real patient case.  Participant research numbers. |
| **Conducting the simulation:** | FULL-SCALE  Location: Apartment on Sykekatu, Lahti, Finland.  Dispatch code: acute severe abdominal pain  Units: EPH 00, EPH 12X & EPH 12X  If the paramedic unit asks for additional information:  ”Elderly woman, who calls for help. The caller related that, one hour ago, she began to experience very severe abdominal pain that radiates to the back. The caller is at home alone and has opened the front door.”  All units arrive on the scene at the same time.  At the scene:  An elderly person lies on the sofa. She immediately complains of an undefined abdominal ache and is unable to describe the nature of the pain more specifically. With the pain scale, the patient indicates 10/10.  Vital signs:  A: Open  B: Respiratory rate is moderate. The person can speak long sentences. The auscultation sounds of the lungs are clean. SpO2 is the actor’s natural level. The respiratory rate is the actor’s natural rate.  C: The actor’s actual values. Rad+/+ Fem +/+. The skin is as the actor’s skin is in reality.  D: The actor’s level of consciousness and findings.  E: Complains of pain with exaggerated gestures. The pain localizes to the lower abdomen and radiates to the backside. The back is painful (if palpated  Treatment:  Excluding serious causes of abdominal pain.  Decision making:  One-unit case. Who is in charge of the scene? How is the leadership distributed among the paramedic unit? Decision making: is this a critically ill patient or not? |
| **Attention:** | **Safety word to stop the simulation: Acute-repeat acute. Everyone shouts acute.**  The simulation scenario can flow spontaneously and take its own course; only the actors will be guided and the prehospital physician and prehospital personnel will be observed for research purposes. |
| **Supplies:** | Paramedic unit equipment X 2  Prehospital physician unit  Personal gear (clothes, work shoes, vests)  iSimulate  VIRVE x 8 (6 for trial subjects)  Connection to Codea control 🡪 Dispatch message  Prop:  Camera/ camera operator  Simulation instructor  Observers  Vest with sign indicating the voluntary outside  defusing professional |
| **Defusing after the simulation:** | After the simulation scenario, a short defusing is held to make sure that everyone is alright. This does not entail any inspection on individual performance. |

**Additional file 8**

**Simulation scenario 3**

| **Target group:** | Päijät-Häme Wellbeing Services County, prehospital physician and paramedics  Control group (5 + 1)  Intervention group (5 + 1) |
| --- | --- |
| **Aim of the simulation:** | The purpose of the simulation is to investigate the stress reactions and actions of prehospital personnel under pressure, as well as their teamwork, ability to maintain situational awareness and decision making.  This the third in a series of three simulation scenarios included in the study “**Effectiveness of Finnish Sisu Training, a pre-emptive resilience coaching programme in enhancing the work performance of prehospital personnel”**. |
| **Preliminary debriefing:** | The study participants taking part in the simulation scenarios are briefed to conduct themselves as if they were involved in a real patient case scene.  Familiarizing the participants with the equipment on hand and making sure that it functions properly.  Checking that the VIRVE radiofrequency channels are correct.  The starting point of the simulation is established, and participants are reminded of the simulation being a part of this research and that the simulation will be a full-scale scenario. The study participant should perform as they would in a real patient case.  Participant research numbers.  Please note! wetsuit = patient’s skin |
| **Conducting the simulation:** | FULL-SCALE  Location: Asikkala  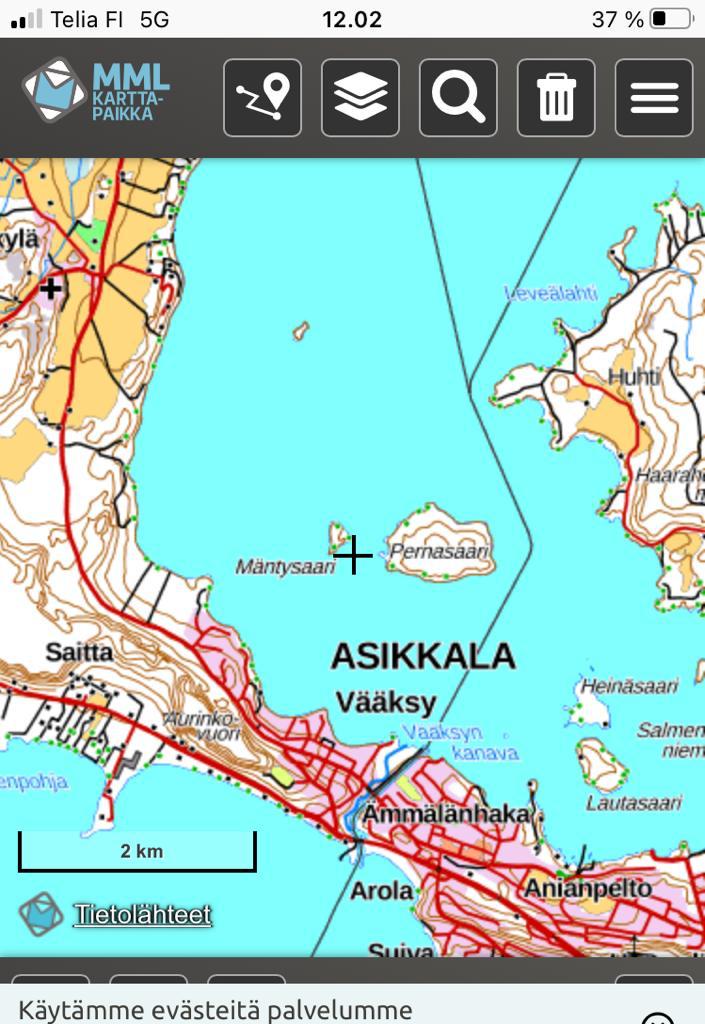  Dispatch code: A221, watercraft accident, small  Units: EPH 00, EPH 225 EPH 226 + lake rescue VPH108  A jet ski has collided with an underwater rock. The driver has been thrown into the water. A distant fisherman has seen the accident and is calling help (112). He does not have the opportunity move closer.  If the paramedic unit asks for additional information:  “The caller has seen and heard from a distance a jet ski crash into an underwater rock. The driver has been flown in an arch into the water. The caller is far away and can’t move closer to see in more detail.”  ”The region’s fire department is occupied with another assignment in Vierumäki. The more remote fire department rescue team unit no. 201 is also occupied.”  EPH00 and EPH225 have just signed off from another assignment in Asikkala (X-5), and EPH226 is travelling back to their base near the scene. The local lake rescue is travelling by boat in the area. The lake rescue suggests that they can pick up the paramedics onboard from the nearby harbour, “Majakka”.  On the scene:  A man is in the water.  Vital signs:  A: Open  B: Respiratory rate is elevated, 25–30 breaths/minute. The patient can speak sentences. SpO2 with a bad curve line, 93%–95%, lung auscultation is clean on both sides.  C: The extremities are s cold due to being in the water. Blood pressure is 186/98 mmHg and heart rate is 110 beats/minute.  D: Oriented, but consciousness fluctuates. On the boat, the patient loses consciousness intermittently. However, he is easily woken up. No differences between pupils. Mimicking is symmetrical. Neurologically, shaking of hands is symmetrical. The lower limbs do not move voluntarily or react in any way. Numbness from waist down. Blood glucose is 6.3 mmol/l. Blood alcohol concentration 0.89 per mille.  E: The thorax is stable, the abdomen is soft and not distended. The scull has a bruise on the right side. The patients complains of a headache. The upper limbs function properly. The lower limbs are not functioning. Ear temperature 33.2 °C.  Treatment:  Transport from water to boat? Emergency transport?  Keeping the patient warm.  Use of prehospital trauma team protocol process. |
| **Attention:** | **Safety word to stop the simulation: Acute-repeat acute. Everyone shouts acute.**  The simulation scenario can flow spontaneously and take its own course; only the actors will be guided and the prehospital physician and prehospital personnel will be observed for research purposes. |
| **Supplies:** | Paramedic unit equipment X 2  Prehospital physician unit  Personal gear (clothes, work shoes, vests)  iSimulate  VIRVE x 8 (6 for trial subjects)  Connection to Codea control 🡪 Dispatch message  Prop:  Camera/ camera operator  Simulation instructor  Observers  Vest with sign indicating the voluntary outside  defusing professional |
| **Defusing after the simulation:** | After the simulation scenario, a short defusing is held to make sure that everyone is alright. This does not entail any inspection on individual performance. |

**Additional file 9**

Table 2. Self-evaluation results.

| Simulation scenario | Before | | p-value | After | | p-value |
| --- | --- | --- | --- | --- | --- | --- |
|  | Intervention group median (IQR) | Control group median (IQR) |  | Intervention group median (IQR) | Control group median (IQR) |  |
| Scenario 1 |  |  |  |  |  |  |
| 1 | 4 (3–4) | 3 (3–4) | 0.59 | 4 (4–4) | 4 (3–4) | 0.23 |
| 2 | 4 (3–4) | 4 (3–5) | 0.98 | 4 (4–4) | 4 (3–4) | 0,49 |
| 3 | 4 (4–4) | 4 (4–4) | >0.99 | 4 (3–5) | 4 (4–4) | >0.99 |
| 4 | 4 (4–4) | 5 (4–5) | 0.12 | 5 (4–5) | 5 (4–5) | >0.99 |
| Scenario 2 |  |  |  |  |  |  |
| 1 | 4 (4–4) | 4 (3–5) | 0.63 | 4 (3–4) | 5 (4–5) | 0.21 |
| 2 | 4 (3–4) | 4 (3–4) | 0.98 | 4 (4–4) | 4 (4–5) | 0.36 |
| 3 | 4 (4–4) | 4 (4–5) | >0.99 | 4 (3–4) | 4 (3–5) | 0.41 |
| 4 | 4 (4–4) | 5 (4–5) | 0.12 | 5 (4–5) | 5 (4–5) | >0.99 |
| Scenario 3 |  |  |  |  |  |  |
| 1 | 4 (3–5) | 4 (3–4) | 0.69 | 5 (4–5) | 4 (3–5) | 0.23 |
| 2 | 4 (4–4) | 4 (4–5) | > 0.99 | 4 (3–4) | 4 (3–5) | 0.41 |
| 3 | 4 (4–5) | 4 (4–4) | >0.99 | 5 (4–5) | 4 (3–5) | 0.12 |
| 4 | 5 (4–5) | 5 (5–5) | 0.28 | 5 (4–5) | 5 (4–5) | >0.99 |

1 = ‘*I feel confident that I will manage the simulation scenario well*; 2 = ‘*I consider myself to have good situational awareness*’; 3 = ‘*I am able to make decisions easily*’; and 4 = ‘*I believe it will be easy to resume other duties after the simulation*’. Likert scale: 1 = very insecure, 2 = mostly insecure, 3 = does not know, 4 = confident, 5 = very confident

**Additional file 10**

Table 3. Comparison of median and interquartile range (IQR) physiological parameters, maximum heart rate and respiratory rate.

|  | n = 8 | n = 8 |  |
| --- | --- | --- | --- |
|  | Intervention group | Control group | p-value |
| Maximum heart rate during simulation scenario (beats/min), median (IQR) |  |  |  |
| 1 | 135 (121–146) | 125 (114–135) | 0.18 |
| 2 | 123 (115–139) | 113 (107–133) | 0.39 |
| 3 | 139 (135–153) | 130 (116–136) | 0.041 |
| Respiratory rate before simulation scenario (breaths/min), median (IQR) |  |  |  |
| 1 | 12 (12–18) | 13 (12–20) | 0.53 |
| 2 | 13 (12–19) | 15 (12–20) | 0.91 |
| 3 | 16 (13–20) | 16 (13–18) | 0.72 |
| Respiratory rate after simulation scenario (breaths/min), median (IQR) |  |  |  |
| 1 | 14 (12–18) | 20 (15–24) | 0.10 |
| 2 | 19 (12–20) | 16 (12–21) | 0.75 |
| 3 | 18 (15–24) | 19 (16–24) | 0.90 |
